# Supplementary material for: Hyperbolic mapping of human proximity networks
Source: Sci Rep. 2020 Nov 20;10:20244. doi: 10.1038/s41598-020-77277-7 (PMC7679465; doi:10.1038/s41598-020-77277-7)
Supplement: Supplementary file 1 — Supplementary Information. [file 41598_2020_77277_MOESM1_ESM.pdf]

Supplementary Information for
Hyperbolic Mapping of Human Proximity Networks

Marco A. Rodríguez-Flores* and Fragkiskos Papadopoulos†
Department of Electrical Engineering, Computer Engineering and Informatics, Cyprus University of Technology, 3036 Limassol, Cyprus

CONTENTS

I. Connection probability in the time-aggregated network 1

II. Inference of latent coordinates with the original and modified Mercator 2

III. Hyperbolic maps of the conference and Friends & Family 3

IV. Human-to-human greedy routing 3

V. Stability of the inferred node coordinates in different days 5

VI. Modified Mercator 7

VII. Aggregation interval and rotation of angular coordinates 9

I. CONNECTION PROBABILITY IN THE TIME-AGGREGATED NETWORK

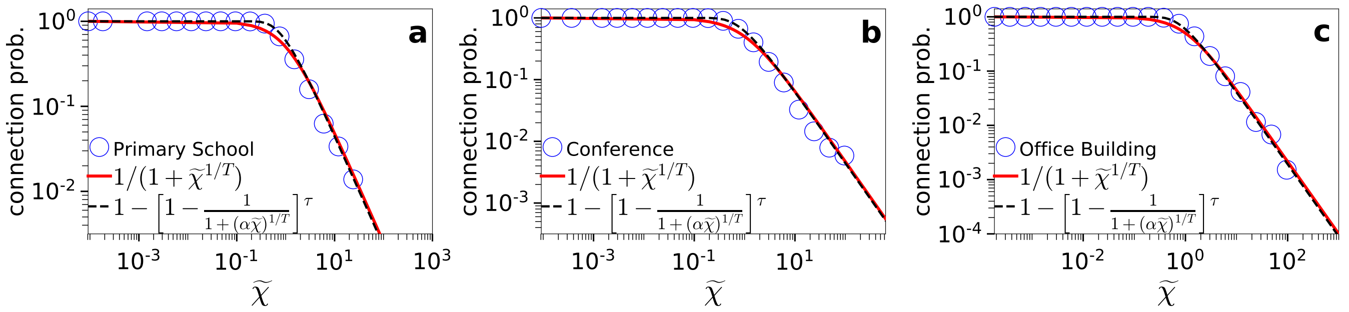

FIG. S1. **Connection probability in the time-aggregated network versus Fermi-Dirac connection probability.** Same as in Fig. 1 in the main text but for the synthetic counterparts of the primary school, conference and office building.

* mj.rodriguezflores@edu.cut.ac.cy
† f.papadopoulos@cut.ac.cy

II. INFERENCE OF LATENT COORDINATES WITH THE ORIGINAL AND MODIFIED MERCATOR

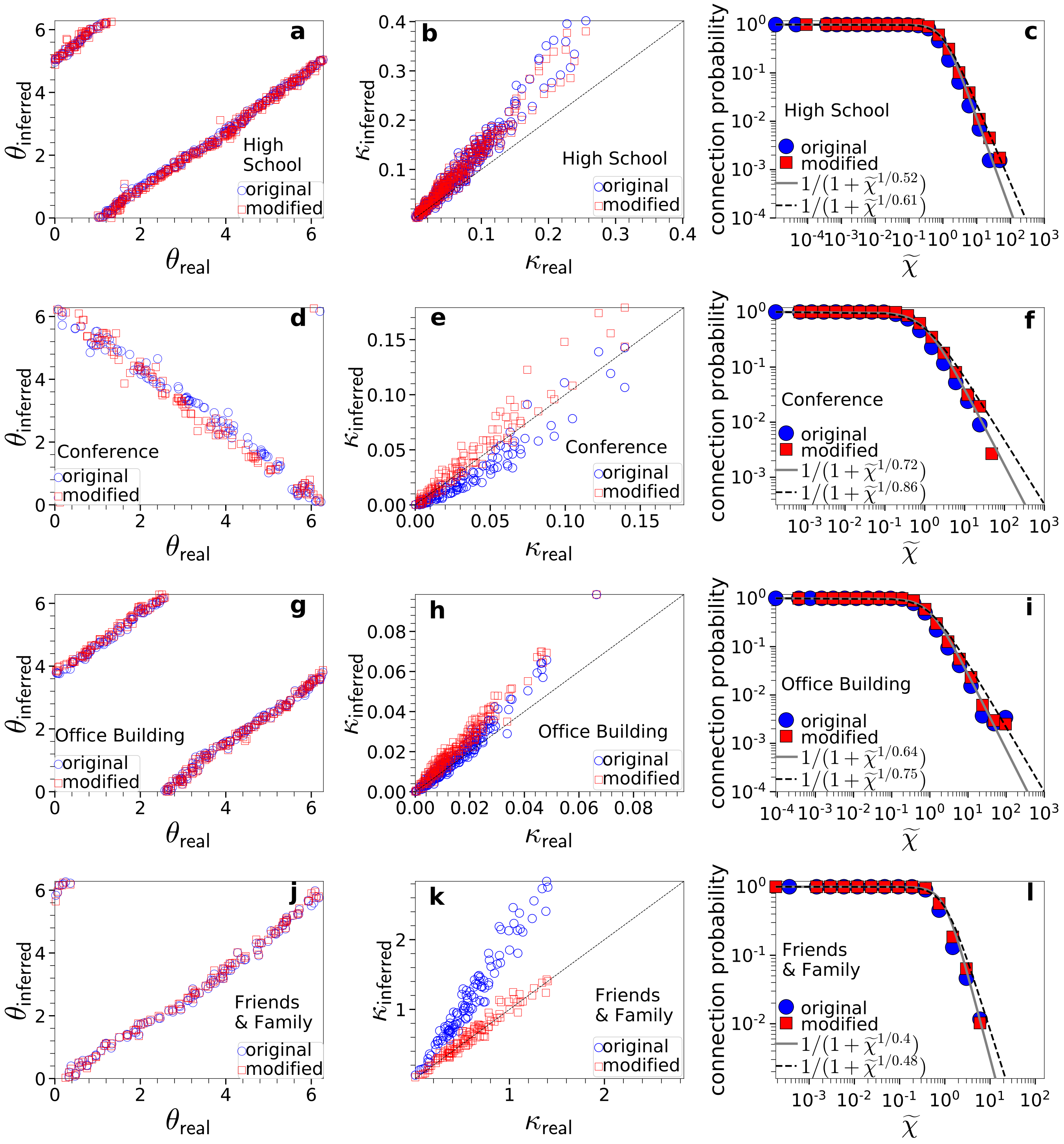

FIG. S2. **Inference of latent coordinates** (κ, θ) **with the original and modified versions of Mercator.** Same as in Fig. 2 in the main text but for the synthetic counterparts of the high school, conference, office building and Friends & Family. For the four networks, the original version estimates $T = 0.52, 0.72, 0.64$ and 0.4, the modified version estimates $T = 0.61, 0.86, 0.75$ and 0.48, while the actual values are $T = 0.61, 0.85, 0.74$ and 0.48, respectively.

III. HYPERBOLIC MAPS OF THE CONFERENCE AND FRIENDS & FAMILY

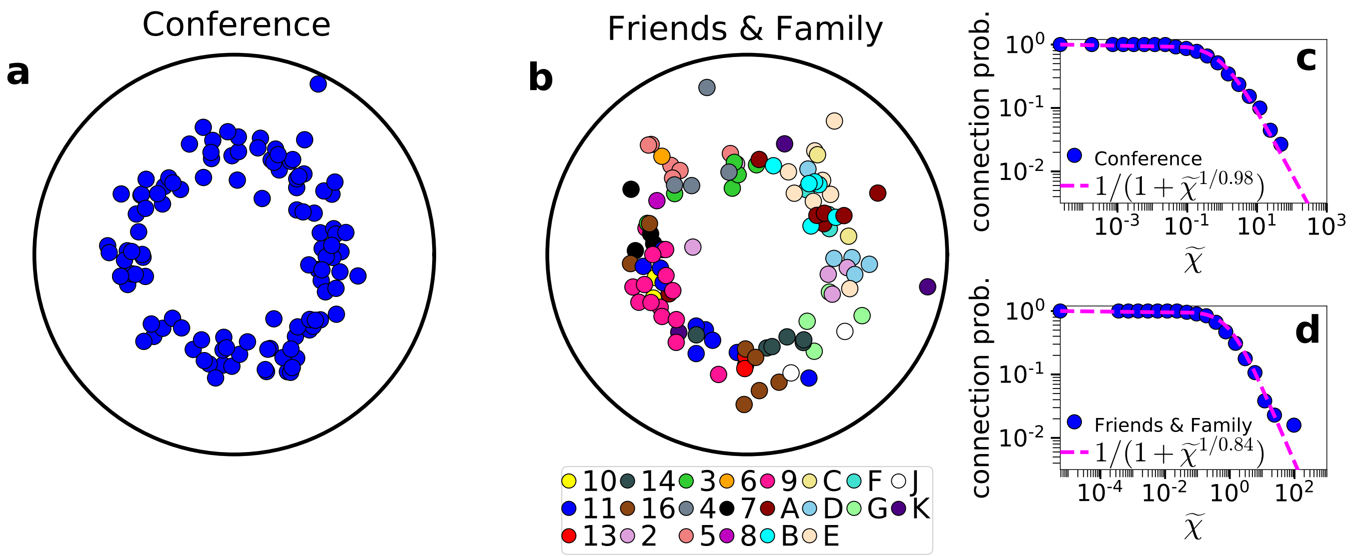

FIG. S3. **Hyperbolic embeddings of the conference and Friends & Family.** Same as in Fig. 4 in the main text but for the conference and Friends & Family. In (**a**) all nodes have the same color as there is no group membership information available for the conference. In (**b**) the nodes are colored according to the partial apartment number or letter where they live, as given in the Friends & Family metadata. The pink dashed lines in (**c**) and (**d**) are Fermi-Dirac connection probabilities with temperatures T as inferred by Mercator, $T = 0.98$ and 0.84, respectively.

IV. HUMAN-TO-HUMAN GREEDY ROUTING

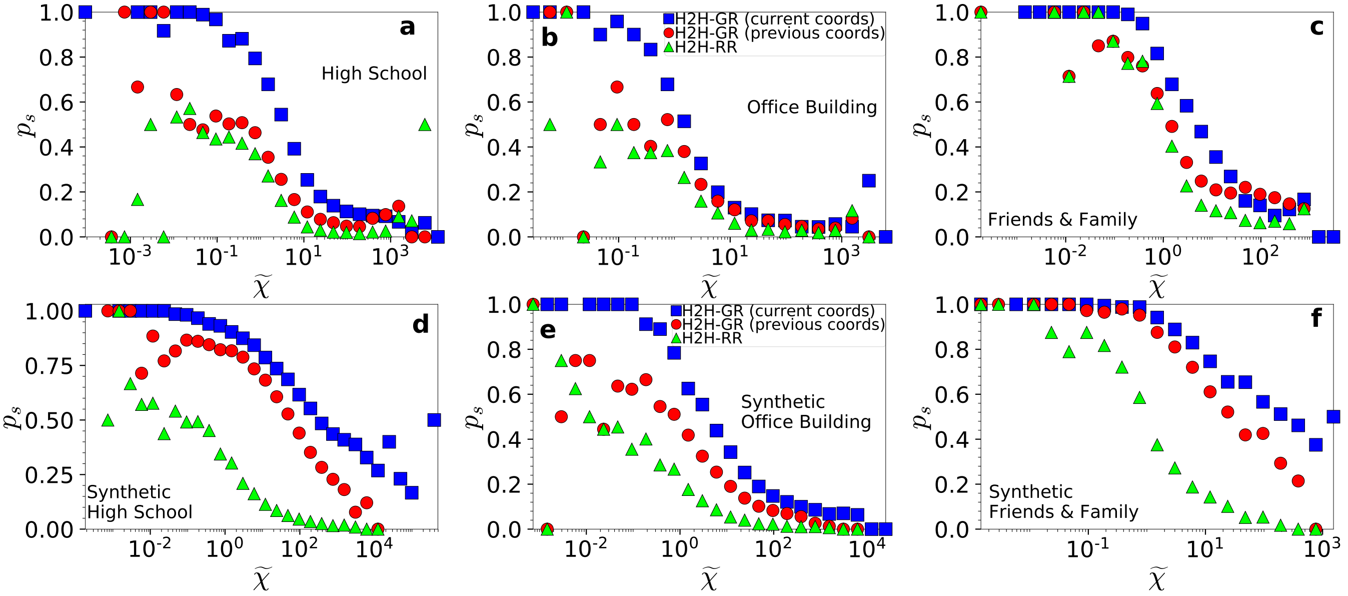

FIG. S4. **Success ratio p_s of H2H-GR and H2H-RR as a function of the effective distance $\tilde{\chi}$ between source-destination pairs.** Same as in Fig. 5 in the main text but for the high school, office building and Friends & Family. The top row shows the results for the real networks, while the bottom row shows the results for the synthetic counterparts.

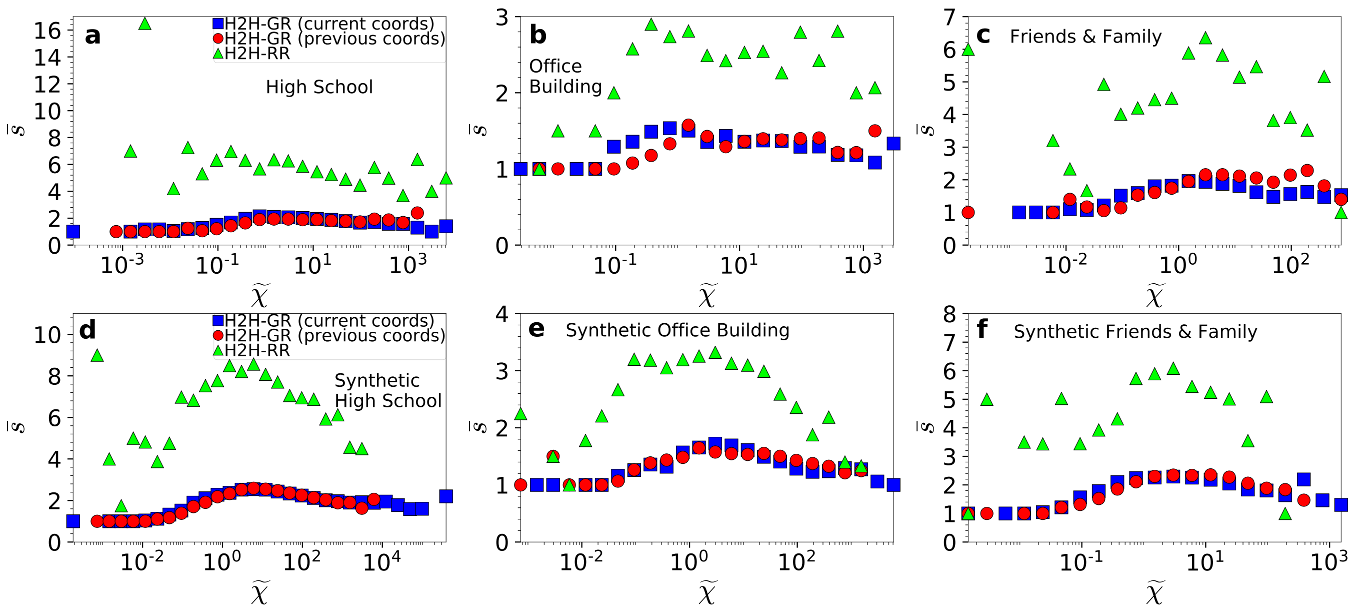

FIG. S5. **Average stretch $\bar{s}$ of H2H-GR and H2H-RR as a function of the effective distance $\tilde{\chi}$ between source-destination pairs.** The results correspond to the networks of Fig. S4.

Real Network	H2H-GR (current angular coordinates)	H2H-GR (previous angular coordinates)
Hospital	$p_s = 0.69$, $\bar{s} = 2.16$	$p_s = 0.39$, $\bar{s} = 1.98$
Primary School	$p_s = 0.69$, $\bar{s} = 4.40$	$p_s = 0.65$, $\bar{s} = 3.88$
Conference	$p_s = 0.55$, $\bar{s} = 2.25$	$p_s = 0.35$, $\bar{s} = 2.11$
High School	$p_s = 0.20$, $\bar{s} = 2.12$	$p_s = 0.10$, $\bar{s} = 1.84$
Office Building	$p_s = 0.11$, $\bar{s} = 1.42$	$p_s = 0.09$, $\bar{s} = 1.39$
Friends & Family	$p_s = 0.42$, $\bar{s} = 2.20$	$p_s = 0.28$, $\bar{s} = 2.03$

TABLE S1. **Success ratio p_s and average stretch $\bar{s}$ of H2H-GR that uses only the angular (similarity) distances in real networks.** Same as in Table 2 in the main text but when using only the inferred angular coordinates (current and previous) in H2H-GR.

Synthetic Network	H2H-GR (current angular coordinates)	H2H-GR (previous angular coordinates)
Hospital	$p_s = 0.71$, $\bar{s} = 2.46$	$p_s = 0.59$, $\bar{s} = 2.44$
Primary School	$p_s = 0.97$, $\bar{s} = 4.77$	$p_s = 0.91$, $\bar{s} = 5.29$
Conference	$p_s = 0.70$, $\bar{s} = 2.83$	$p_s = 0.51$, $\bar{s} = 2.77$
High School	$p_s = 0.35$, $\bar{s} = 2.93$	$p_s = 0.25$, $\bar{s} = 2.90$
Office Building	$p_s = 0.13$, $\bar{s} = 1.66$	$p_s = 0.10$, $\bar{s} = 1.61$
Friends & Family	$p_s = 0.58$, $\bar{s} = 2.58$	$p_s = 0.49$, $\bar{s} = 2.47$

TABLE S2. Same as in Table S1 but for the synthetic counterparts of the real systems.

V. STABILITY OF THE INFERRED NODE COORDINATES IN DIFFERENT DAYS

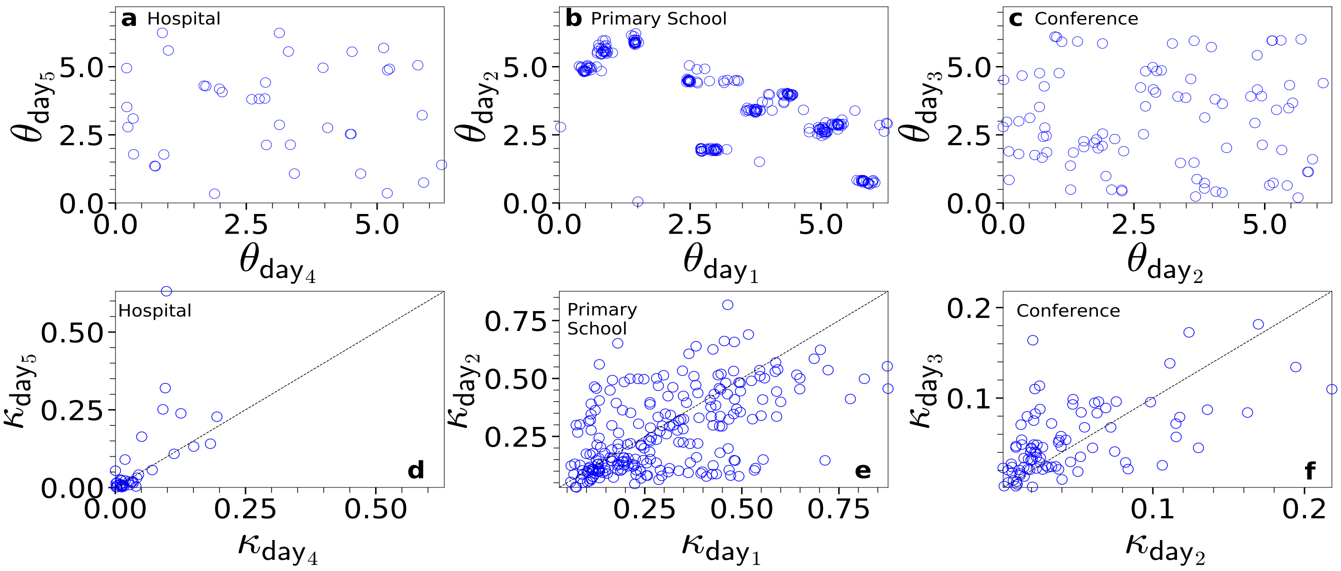

FIG. S6. **Inferred node coordinates (κ, θ) from the time-aggregated network of different observation days.** The results correspond to real networks, and the considered days are as in Table 2 in the main text. (**a**) Inferred angles in day 4 versus inferred angles in day 5 in the hospital. The numbers of time slots for days 4 and 5 are $\tau = 3889$ and 2177, respectively, while Mercator's inferred temperature for the time-aggregated network is $T = 0.99$ for both days. (**b**) Inferred angles in day 1 versus inferred angles in day 2 in the primary school. For days 1, 2, $\tau = 1555, 1545$ and $T = 0.43, 0.36$. (**c**) Inferred angles in day 2 versus inferred angles in day 3 in the conference. For days 2, 3, $\tau = 3216, 1946$ and $T = 0.99, 0.98$. (**d**-**f**) Same as in (**a**-**c**) but for the inferred latent degrees κ. For each node, κ is estimated as $\kappa = \tilde{\kappa}/\alpha$, where $\tilde{\kappa}$ is the node's inferred latent degree in the time-aggregated network of the corresponding day, while $\alpha = \tau^T/\Gamma(1+T)$. Due to rotational symmetry of the model, the inferred angles in a day can be globally shifted compared to the inferred angles in another day by any value in $[0, 2\pi]$.

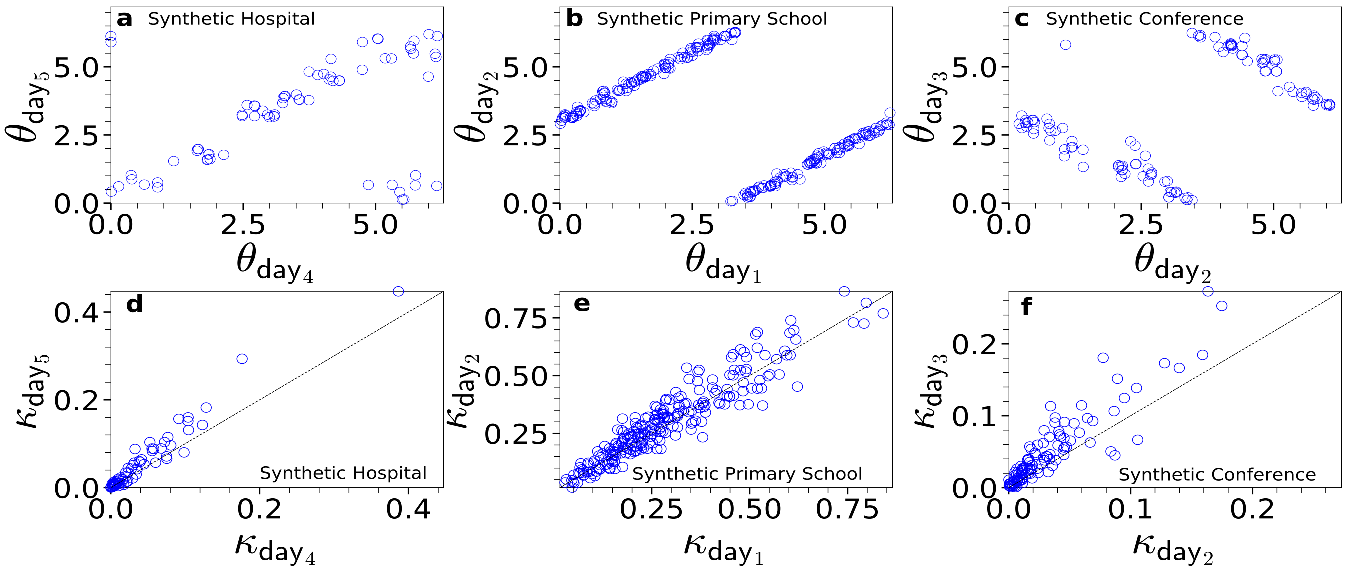

FIG. S7. **Inferred node coordinates (κ, θ) from the time-aggregated network of different days.** Same as in Fig. S6 but for the synthetic counterparts of the real systems. The days in each counterpart have the same duration τ as in the corresponding real system. The temperatures inferred by Mercator are $T = 0.57$ for both days of the hospital, $T = 0.60, 0.64$ for days 1, 2 of the primary school, and $T = 0.64$ for both days of the conference.

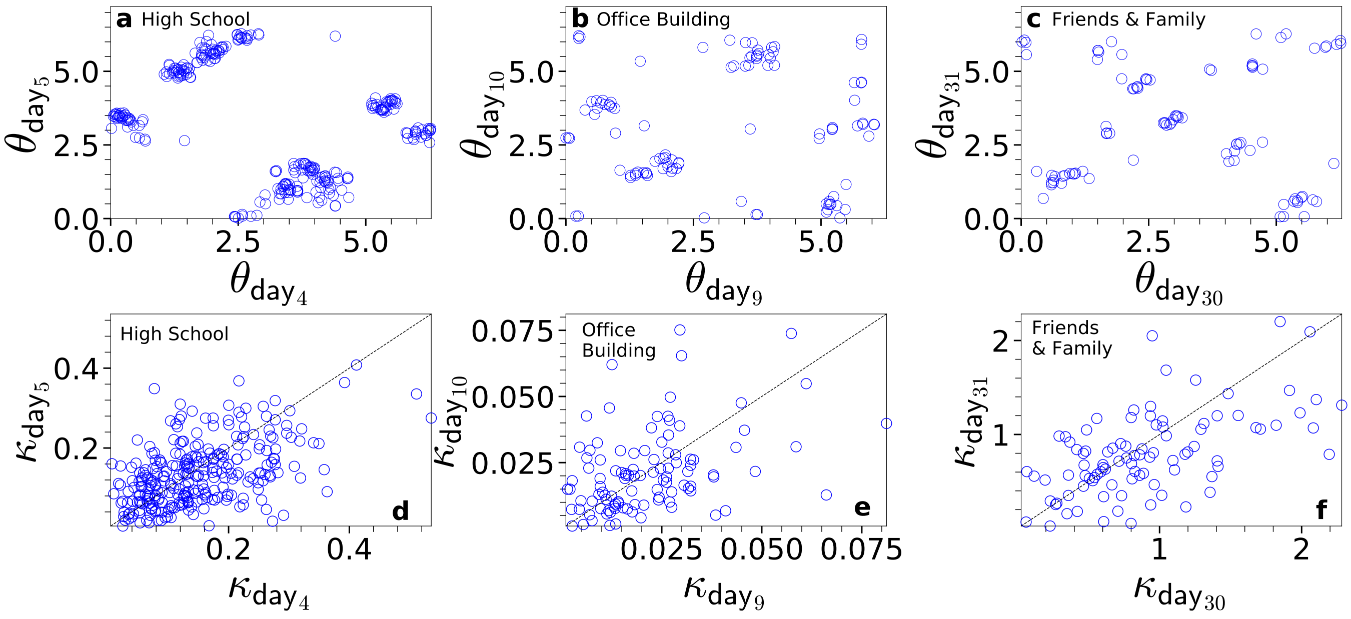

FIG. S8. **Inferred node coordinates** (κ, θ) **from the time-aggregated network of different observation days.** Same as in Fig. S6 but for the high school, office building and Friends & Family. (**a**) Inferred angles in day 4 versus inferred angles in day 5 in the high school. For days $4, 5$, $\tau = 1619$ and $T = 0.54, 0.49$. (**b**) Inferred angles in day 9 versus inferred angles in day 10 in the office building. For days $9, 10$, $\tau = 2153, 2148$ and $T = 0.57, 0.47$. (**c**) Inferred angles in the 30th of March, 2011 versus inferred angles in the 31st of March, 2011 in the Friends & Family. For the two days, $\tau = 289$ and $T = 0.52, 0.49$. (**d-f**) Same as in (**a-c**) but for the inferred latent degrees κ.

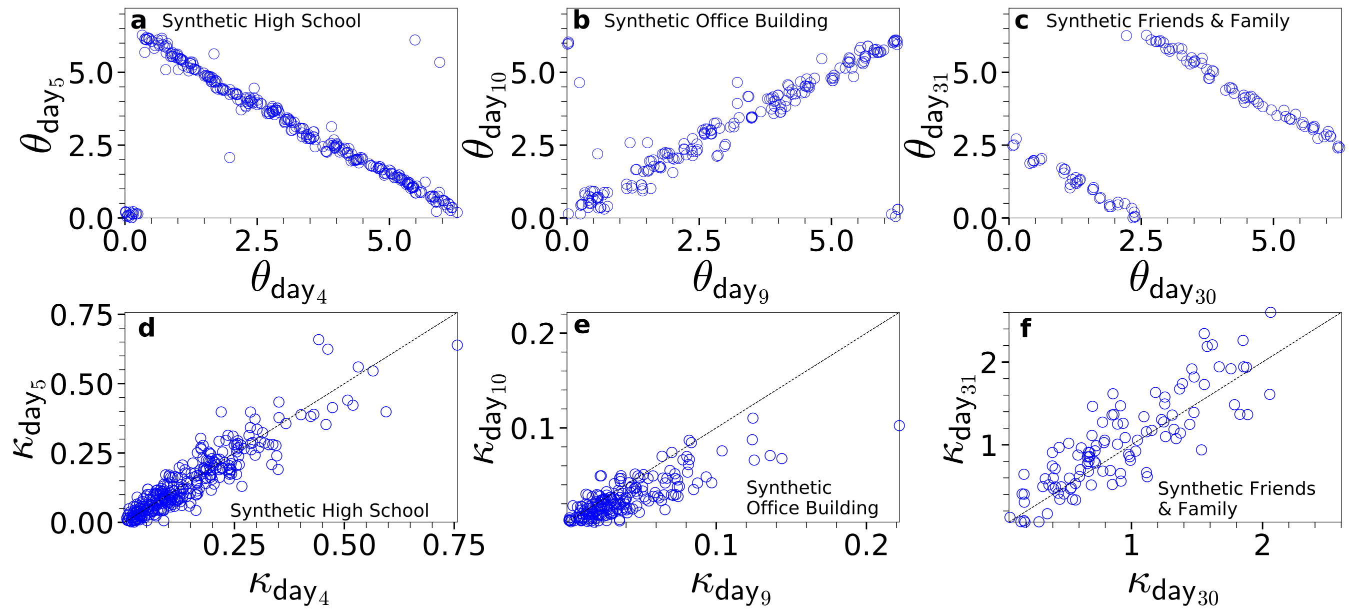

FIG. S9. **Inferred node coordinates** (κ, θ) **from the time-aggregated network of different days.** Same as in Fig. S8 but for the synthetic counterparts of the real systems. The days in each counterpart have the same duration τ as in the corresponding real system. The temperatures inferred by Mercator are $T = 0.54$ for both days of the high school, $T = 0.68$ for both days of the office building, and $T = 0.45, 0.43$ for the two days of the Friends & Family.

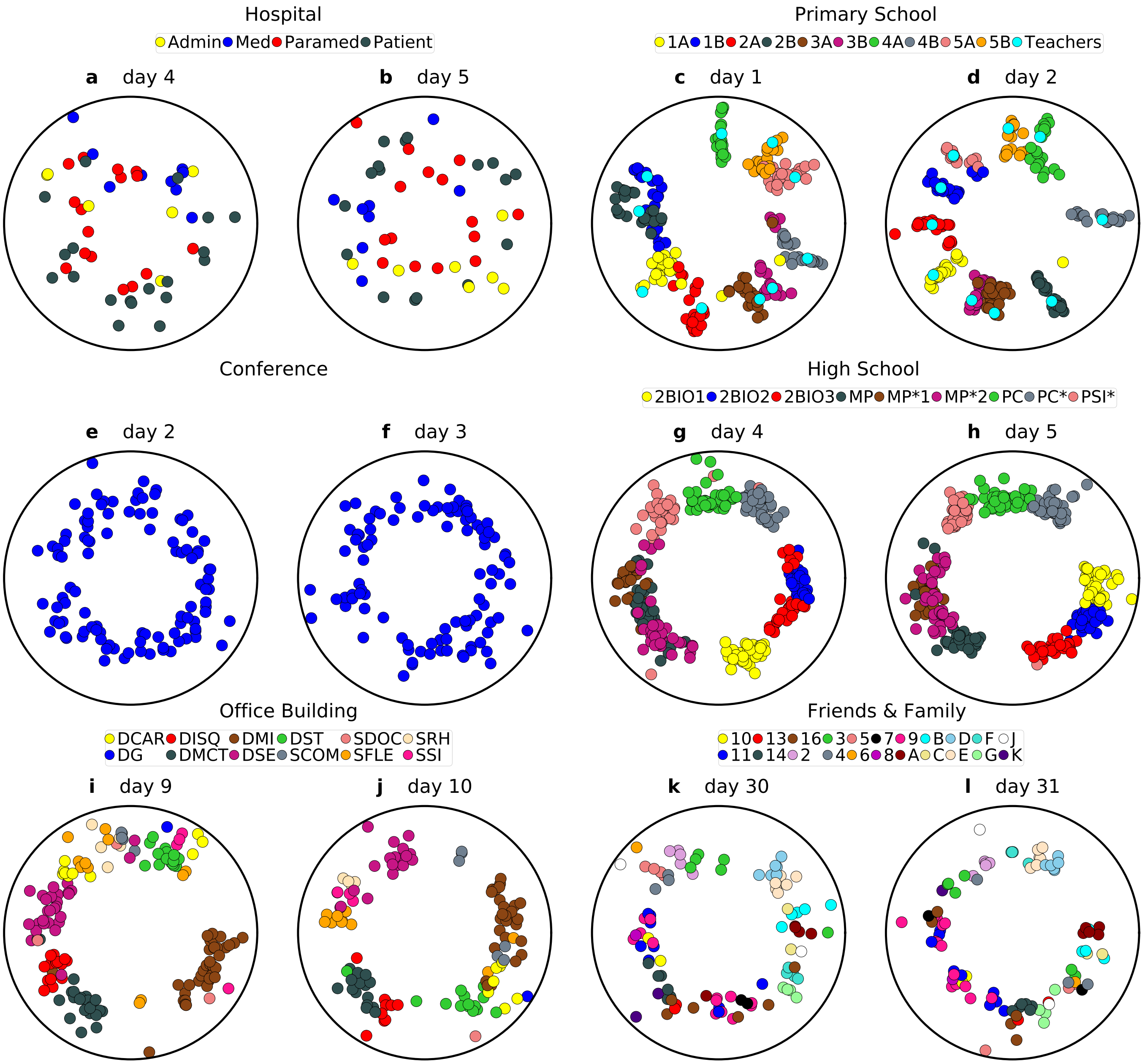

FIG. S10. **Daily hyperbolic maps of the considered real systems.** The maps correspond to the days considered in Figs. S6 and S8. The nodes are positioned according to their inferred hyperbolic coordinates (r, θ) in the time-aggregated network of the corresponding day. For an easier inspection of how node coordinates change between days, the map of each day is rotated such that it minimizes the sum of the squared distances between the inferred angles in the day and the angles inferred by considering the full duration of the corresponding network (Fig. 4 in the main text and Fig. S3). The nodes are colored according to group membership information available in the metadata of each network as described in Fig. 4 of the main text and in Fig. S3.

VI. MODIFIED MERCATOR

In the modified version of Mercator we replace the connection probability of the $\mathbb{S}^1$ model [Eq. (1) in the main text] with the connection probability in the time-aggregated network of the dynamic-$\mathbb{S}^1$ model [Eq. (7) in the main text]. This modification requires replacing all relations in the original Mercator implementation, which are derived using the original connection probability, with the corresponding relations derived using the new connection probability. Below we list all modifications made in each step of the original Mercator implementation (Ref. [23] in the main text).

For convenience, we express all new relations in terms of the nodes' latent degrees per snapshot κ. We recall that $\kappa = \tilde{\kappa}/\alpha$, where $\tilde{\kappa}$ denotes the node's latent degree in the time-aggregated network, while $\alpha = \tau^T/\Gamma(1+T)$. The modified Mercator takes also as input the value of the duration τ in which the time-aggregated network is computed, while like the original version it infers the value of the temperature parameter T along with the nodes' coordinates $(\tilde{\kappa}, \theta)$.

In the first step, Mercator uses an iterative procedure that adjusts the nodes' latent degrees so that the expected degree of each node as prescribed by the $\mathbb{S}^1$ model matches the node's observed degree in the given network. We adapt this step by replacing the relation for the probability that two nodes with latent degrees κ and κ' are connected [Eq. (A1) in Ref. [23] of the main text], with

$$p(a_{\kappa\kappa'}=1) = 1 - \frac{2\mu\kappa\kappa'}{N}\left[\frac{T\Gamma(\tau+T)\Gamma(-T)}{\Gamma(\tau)} + \left(\frac{1}{1+\left(\frac{N}{2\mu\kappa\kappa'}\right)^{1/T}}\right)^{-T} {}_2F_1\left(-T, 1-\tau-T; 1-T; \frac{1}{1+\left(\frac{N}{2\mu\kappa\kappa'}\right)^{1/T}}\right)\right]. \tag{1}$$

The above relation is derived in Ref. [22] of the main text. $a_{\kappa\kappa'}$ is an indicator function, $a_{\kappa\kappa'} = 1$ if two nodes with latent degrees κ and κ' are connected in the time-aggregated network, and $a_{\kappa\kappa'} = 0$ otherwise, $\mu = \sin(T\pi)/(2\bar{\kappa}T\pi)$, and ${}_2F_1(a, b; c; z)$ is the Gauss hypergeometric function.

In the second step, Mercator uses an iterative procedure that adjusts the temperature T so that the value of the average clustering coefficient as prescribed by the $\mathbb{S}^1$ model matches the value of the average clustering coefficient in the given network. We adapt this step by replacing the relation for the distribution of the angular distance $\Delta\theta$ between two connected nodes with latent degrees κ and κ' [Eq. (A3) in Ref. [23] of the main text], with

$$\begin{aligned}\rho(\Delta\theta|a_{\kappa\kappa'}=1) &= \frac{p(a_{\kappa\kappa'}=1|\Delta\theta)\rho(\Delta\theta)}{p(a_{\kappa\kappa'}=1)}\\ &= \frac{\frac{1}{\pi}\left[1-\left(1-\frac{1}{1+\left(\frac{R\Delta\theta}{\mu\kappa\kappa'}\right)^{1/T}}\right)^{\tau}\right]}{1-\frac{2\mu\kappa\kappa'}{N}\left[\frac{T\Gamma(\tau+T)\Gamma(-T)}{\Gamma(\tau)} + \left(\frac{1}{1+\left(\frac{N}{2\mu\kappa\kappa'}\right)^{1/T}}\right)^{-T} {}_2F_1\left(-T, 1-\tau-T; 1-T; \frac{1}{1+\left(\frac{N}{2\mu\kappa\kappa'}\right)^{1/T}}\right)\right]}.\end{aligned} \tag{2}$$

In the above relation, $p(a_{\kappa\kappa'} = 1|\Delta\theta)$ is the probability that two nodes with latent degrees κ and κ' and angular distance $\Delta\theta$ are connected in the time-aggregated network, $\rho(\Delta\theta) = 1/\pi$ is the uniform distribution of the angular distances in the model, and $p(a_{\kappa\kappa'} = 1)$ is given by (1).

In the third step, Mercator adapts Laplacian Eigenmaps (LE) to the $\mathbb{S}^1$ model in order to determine initial angular coordinates for the nodes. We adapt this step by replacing the relation for the expected angular distance between two nodes with latent degrees κ_i and κ_j conditioned on the fact that they are connected [Eq. (A8) in Ref. [23] of the main text], with

$$\begin{aligned}\langle\Delta\theta_{ij}\rangle &= \int_0^{\pi}\Delta\theta_{ij}\rho(\Delta\theta_{ij}|a_{\kappa_i\kappa_j}=1)\mathrm{d}\Delta\theta_{ij}\\ &= \frac{N\pi\Gamma(\tau)\left[\tau+2T-2T\left(\frac{\pi R}{\mu\kappa_i\kappa_j}\right)^{\tau/T} {}_2F_1\left(\tau, \tau+2T; \tau+2T+1; -\left(\frac{\pi R}{\mu\kappa_i\kappa_j}\right)^{1/T}\right)\right]}{2(\tau+2T)\left[\left(N+2T\mu\kappa_i\kappa_j\mathrm{B}(x; -T, \tau+T)\right)\Gamma(\tau)+2\mu\kappa_i\kappa_j\Gamma(1-T)\Gamma(\tau+T)\right]},\end{aligned} \tag{3}$$

where $\mathrm{B}(x; a, b)$ is the incomplete beta function and $x = 1/\left[1+\left(\frac{N}{2\mu\kappa_i\kappa_j}\right)^{1/T}\right]$. In this step, we also replace the probability in the $\mathbb{S}^1$ model of having the observed connection (or disconnection) among each pair of consecutive nodes i and $i+1$ on the similarity circle, conditioned on their angular separation gap g_i and their latent degrees κ_i and κ_j [Eq. (A13) in Ref. [23] of the main text], with

$$p(a_{i+1,i}|g_i) = \left[1-\left(1-\frac{1}{1+\left(\frac{Rg_i}{\mu\kappa_i\kappa_j}\right)^{1/T}}\right)^{\tau}\right]^{a_{i+1,i}} \times \left[\left(1-\frac{1}{1+\left(\frac{Rg_i}{\mu\kappa_i\kappa_j}\right)^{1/T}}\right)^{\tau}\right]^{1-a_{i+1,i}}, \tag{4}$$

where $a_{i,i+1} = 1$ if the two nodes are connected in the time-aggregated network, and $a_{i,i+1} = 0$ otherwise.

In the fourth step, Mercator refines the initial angular coordinates by (approximately) maximizing the likelihood that the given network is produced by the $\mathbb{S}^1$ model. We adapt this step by replacing the local log-likelihood for each node i [Eq. (A20) in Ref. [23] of the main text], with

$$\ln \mathcal{L}_i = \sum_{j \neq i} a_{ij} \ln \left[1 - \left(1 - \frac{1}{1 + \left(\frac{R \Delta\theta_{ij}}{\mu \kappa_i \kappa_j} \right)^{1/T}} \right)^{\tau} \right] + (1 - a_{ij}) \ln \left[\left(1 - \frac{1}{1 + \left(\frac{R \Delta\theta_{ij}}{\mu \kappa_i \kappa_j} \right)^{1/T}} \right)^{\tau} \right], \tag{5}$$

where $a_{ij} = 1$ if nodes i and j are connected in the time-aggregated network, and $a_{ij} = 0$ otherwise.

In the final (optional) step, Mercator re-adjusts the latent degrees of the nodes according to the inferred angular coordinates so that the expected degree of each node indeed matches its observed degree in the given network. We adapt this step by replacing the connection probability of the $\mathbb{S}^1$ model in Eq. (A21) in Ref. [23] of the main text, with the connection probability in the time-aggregated network of the dynamic-$\mathbb{S}^1$ model [Eq. (7) in the main text].

VII. AGGREGATION INTERVAL AND ROTATION OF ANGULAR COORDINATES

In Fig. 3 of the main text we quantify the difference between inferred and real coordinates as a function of the aggregation interval τ in a synthetic counterpart of the primary school. In this section, Figs. S11-S15 correspond to the same results but for synthetic counterparts of the hospital, conference, high school, office building and Friends & Family. Specifically, each of Figs. S11-S15 shows the metrics $D_\kappa(\tau)$, $D_\theta(\tau)$ and $d(\tau)$ defined in the caption of Fig. 3 in the main text. Further, Figs. S16-S27 juxtapose the inferred against the real coordinates in each synthetic counterpart, as a function of the aggregation interval τ.

As mentioned in the main text, before computing $D_\theta(\tau)$, we globally shift (rotate) the inferred angles such that the sum of the squared distances (SSD) between real and rotated inferred angles is minimized. To this end, we apply a Procrustean rotation (Ref. [40] in the main text), as follows:

1. We transform the real and inferred angles $\{\theta^i_{\text{real}}\}$ and $\{\theta^i_{\text{inferred}}\}$ to Cartesian coordinates $\{x_i, y_i\} = \{\cos\theta^i_{\text{real}}, \sin\theta^i_{\text{real}}\}$ and $\{w_i, z_i\} = \{\cos\theta^i_{\text{inferred}}, \sin\theta^i_{\text{inferred}}\}$ for all nodes $i = 1, \ldots, N$.[1]

2. A rotation of the points $\{w_i, z_i\}$ by an angle ϕ is given by $\{u_i, v_i\} = \{w_i \cos\phi - z_i \sin\phi, w_i \sin\phi + z_i \cos\phi\}$, where u_i, v_i are the coordinates of the rotated point w_i, z_i. The SSD between $\{u_i, v_i\}$ and $\{x_i, y_i\}$ is SSD $= \sum_{i=1}^N (u_i - x_i)^2 + (v_i - y_i)^2$. The optimal rotation angle ϕ^* is computed by taking the derivative of the SSD with respect to ϕ and solving for ϕ when the derivative is zero,

$$\phi^* = \tan^{-1} \left(\frac{\sum_{i=1}^N (w_i y_i - z_i x_i)}{\sum_{i=1}^N (w_i x_i + z_i y_i)} \right). \tag{6}$$

   We compute the optimally rotated inferred angles as $\{\theta^i_{\text{rotated}}\} = \{\tan^{-1}(v_i^*/u_i^*)\}$, where $\{u_i^*, v_i^*\} = \{w_i \cos\phi^* - z_i \sin\phi^*, w_i \sin\phi^* + z_i \cos\phi^*\}$.[2]

3. We repeat the above procedure after replacing $\{\theta^i_{\text{inferred}}\}$ with $\{2\pi - \theta^i_{\text{inferred}}\}$, which is the reflection of the former across the x-axis, and compute the optimally rotated inferred angles in this case as well, $\{\tilde{\theta}^i_{\text{rotated}}\}$.

4. We compute $D_\theta(\tau) = \sum_{i=1}^N |\theta^i_{\text{rotated}} - \theta^i_{\text{real}}|/N$ and $\widetilde{D}_\theta(\tau) = \sum_{i=1}^N |\tilde{\theta}^i_{\text{rotated}} - \theta^i_{\text{real}}|/N$. The optimally rotated inferred angles are $\{\theta^i_{\text{rotated}}\}$ if $D_\theta(\tau) < \widetilde{D}_\theta(\tau)$, and $\{\tilde{\theta}^i_{\text{rotated}}\}$ otherwise.

We follow a similar procedure for the rotations in Fig. S10.

---

[1] Notation "{ }" denotes a set. For example, $\{x_i, y_i\} = \{x_1, y_1, x_2, y_2, \ldots, x_N, y_N\}$.
[2] If $\theta^i_{\text{rotated}} < 0$, then $\theta^i_{\text{rotated}} := 2\pi + \theta^i_{\text{rotated}}$.

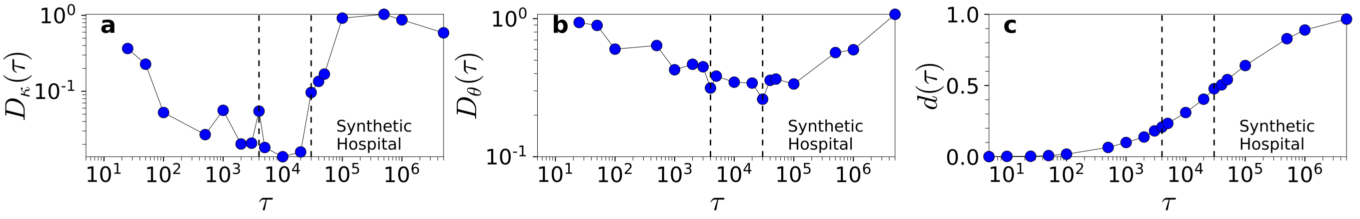

FIG. S11. **Inference accuracy vs. aggregation interval.** Same as in Fig. 3 in the main text but for a synthetic counterpart of the hospital. The vertical dashed lines indicate the interval $4000 \leq \tau \leq 30000$. In this interval, $D_\kappa(\tau) < 0.1$, $D_\theta(\tau) < 0.4$, and $0.20 < d(\tau) < 0.48$.

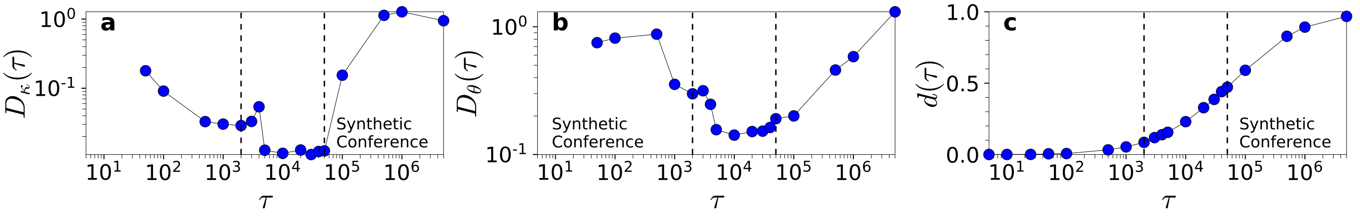

FIG. S12. **Inference accuracy vs. aggregation interval.** Same as in Fig. S11 but for a synthetic counterpart of the conference. The vertical dashed lines indicate the interval $2000 \leq \tau \leq 50000$. In this interval, $D_\kappa(\tau) < 0.1$, $D_\theta(\tau) < 0.4$, and $0.09 < d(\tau) < 0.47$.

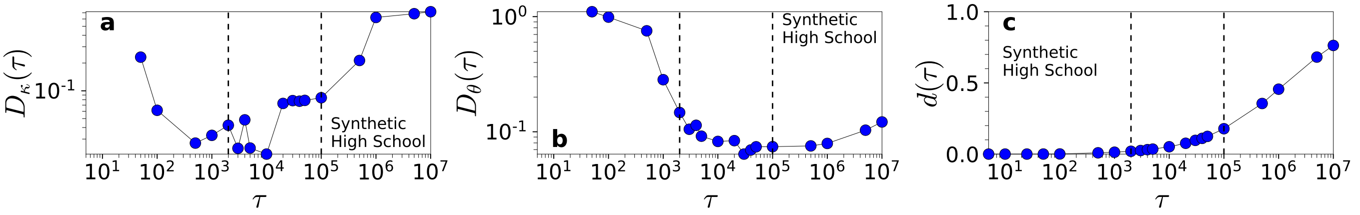

FIG. S13. **Inference accuracy vs. aggregation interval.** Same as in Fig. S11 but for a synthetic counterpart of the high school. The vertical dashed lines indicate the interval $2000 \leq \tau \leq 100000$. In this interval, $D_\kappa(\tau) < 0.1$, $D_\theta(\tau) < 0.2$, and $0.01 < d(\tau) < 0.18$.

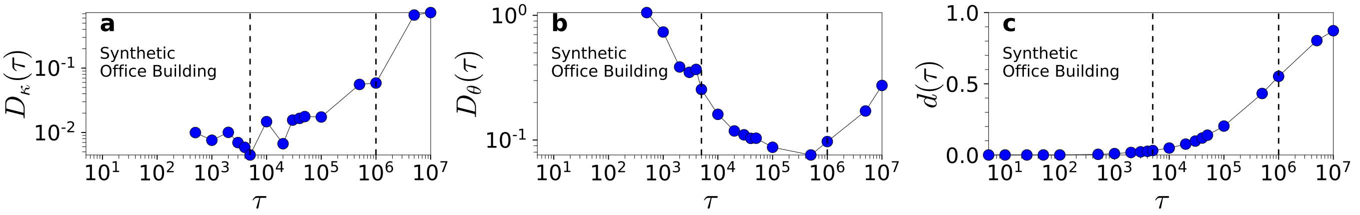

FIG. S14. **Inference accuracy vs. aggregation interval.** Same as in Fig. S11 but for a synthetic counterpart of the office building. The vertical dashed lines indicate the interval $5000 \leq \tau \leq 1000000$. In this interval, $D_\kappa(\tau) < 0.1$, $D_\theta(\tau) < 0.3$, and $0.03 < d(\tau) < 0.55$.

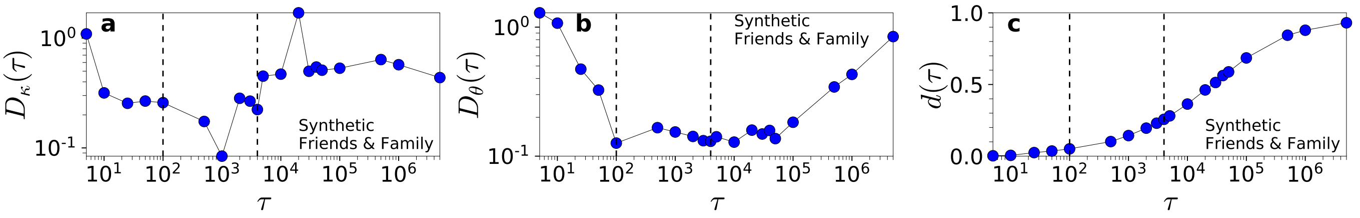

FIG. S15. **Inference accuracy vs. aggregation interval.** Same as in Fig. S11 but for a synthetic counterpart of the friends & family. The vertical dashed lines indicate the interval $100 \leq \tau \leq 4000$. In this interval, $D_\kappa(\tau) < 0.3$, $D_\theta(\tau) < 0.2$, and $0.05 < d(\tau) < 0.26$.

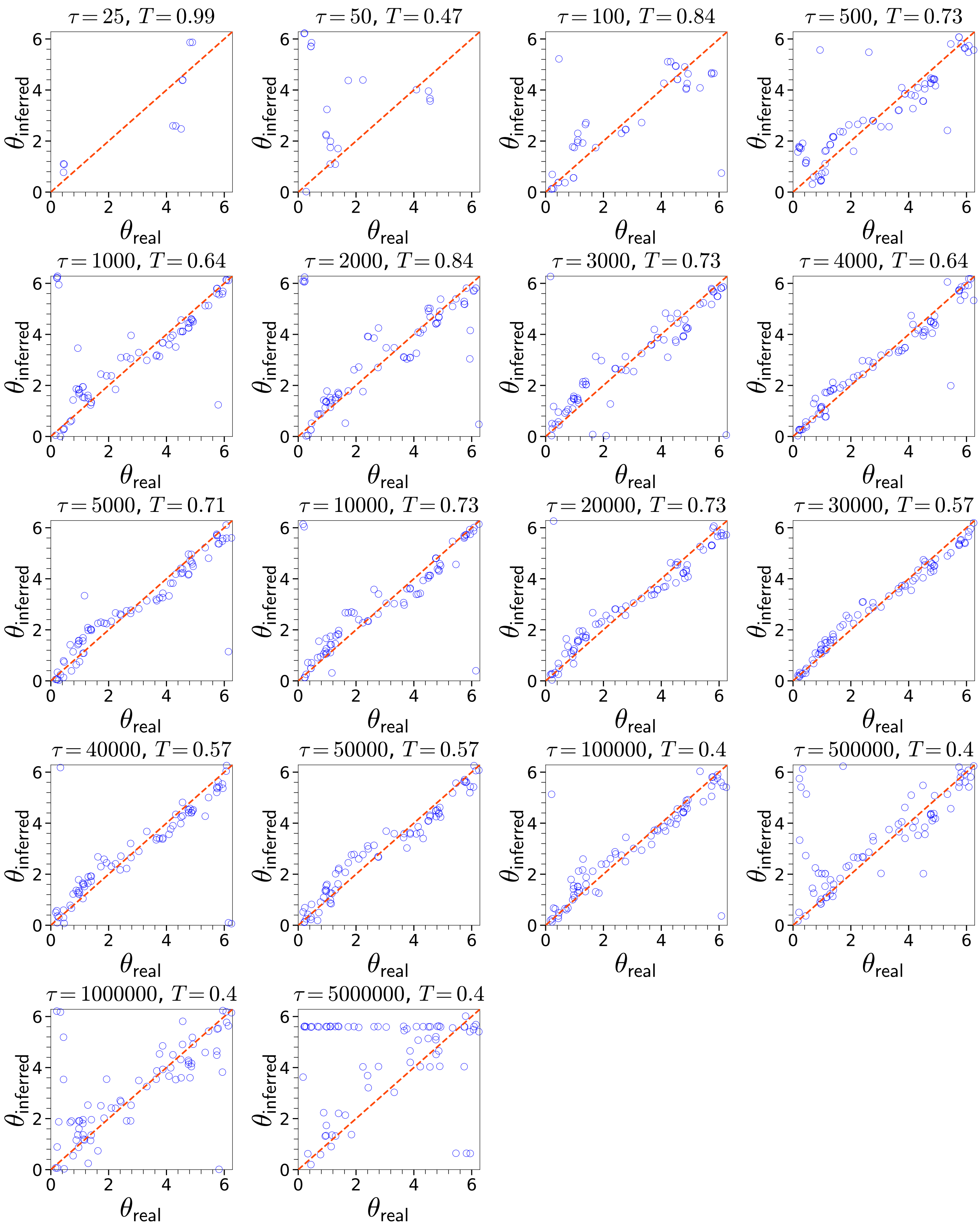

FIG. S16. **Inferred vs. real θ for different aggregation intervals τ.** The results correspond to the synthetic counterpart of the hospital. For each τ we also indicate the temperature T inferred by Mercator. The diagonal dashed line indicates $x = y$.

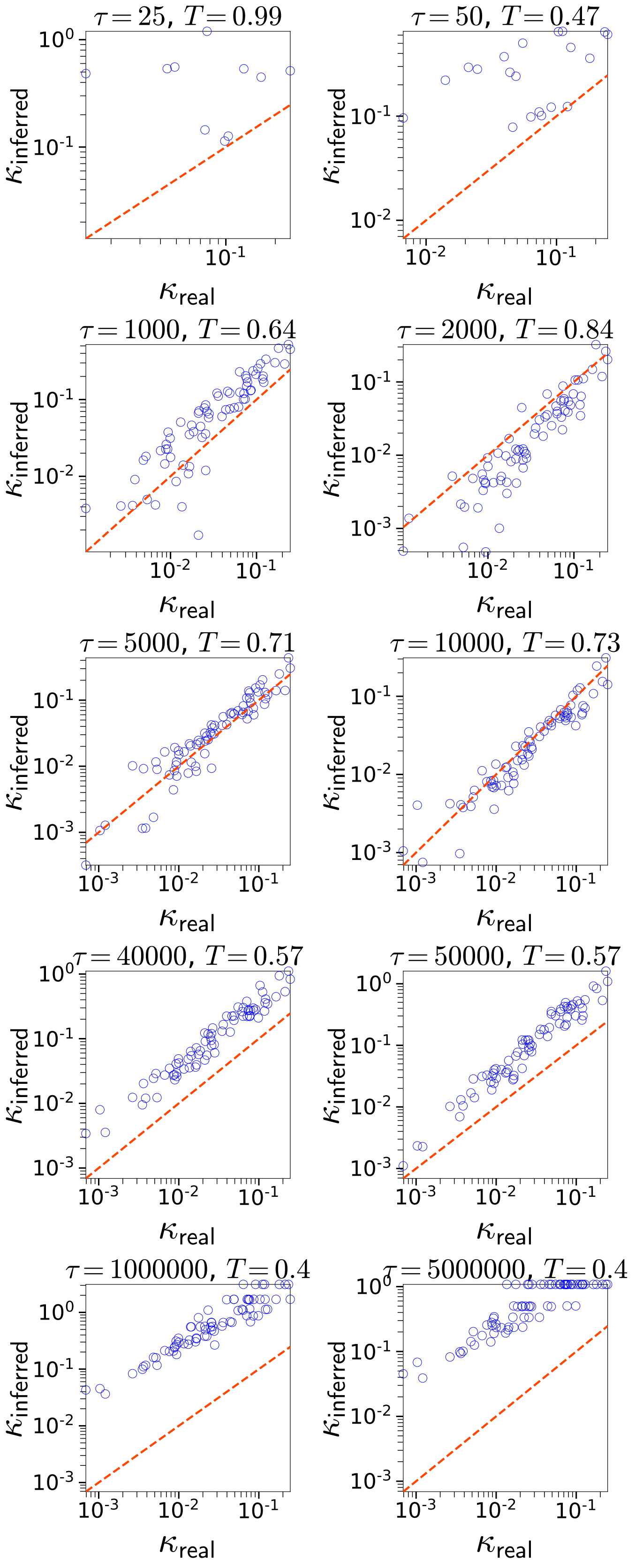

FIG. S17. **Inferred vs. real κ for different aggregation intervals τ.** The results correspond to the synthetic counterpart of the hospital. The κ_{inferred} are estimated as described in the caption of Fig. 2 in the main text. For each τ we indicate the temperature T inferred by Mercator. The diagonal dashed line indicates $x = y$.

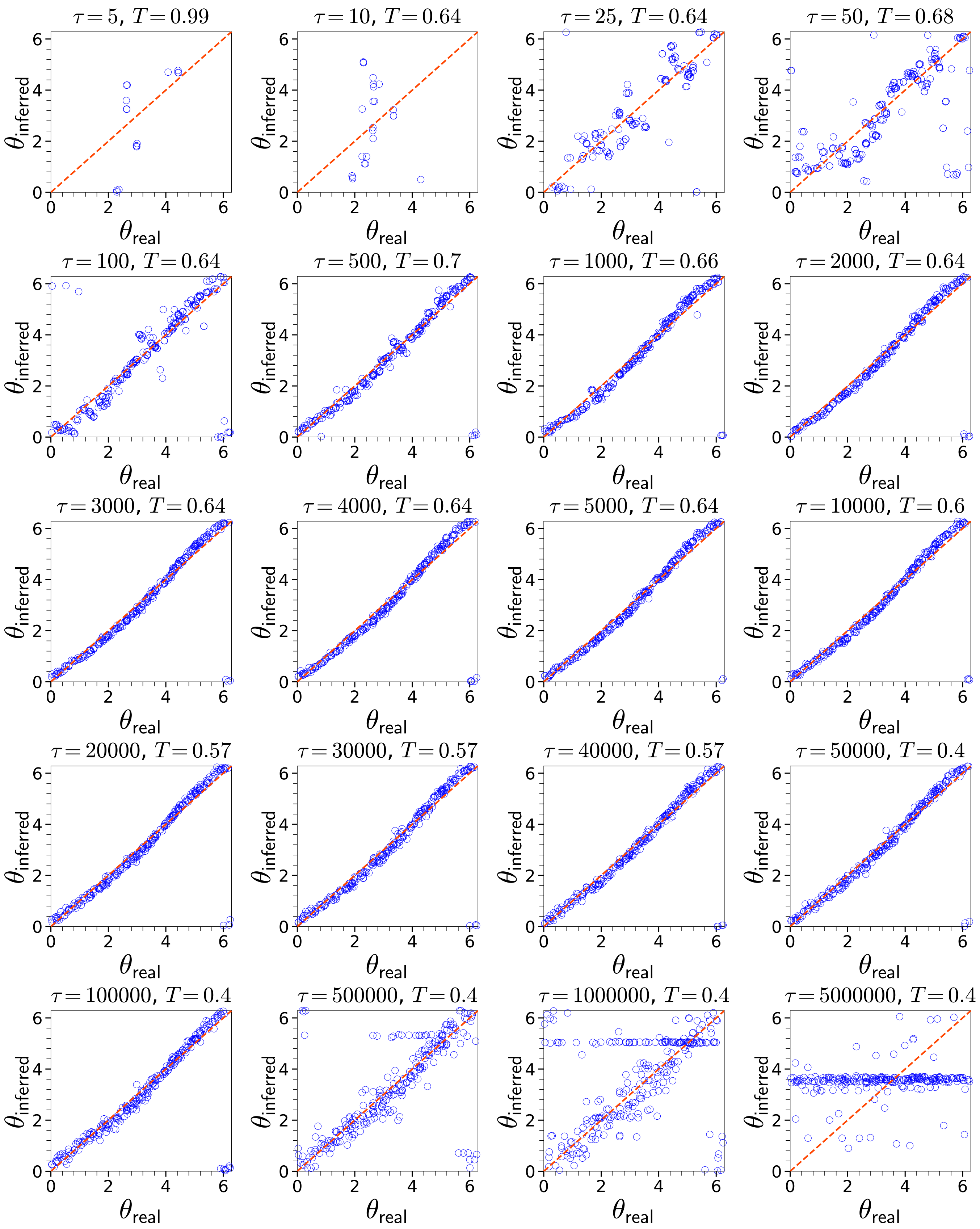

FIG. S18. **Inferred vs. real θ for different aggregation intervals τ.** Same as in Fig. S16 but for the synthetic counterpart of the primary school.

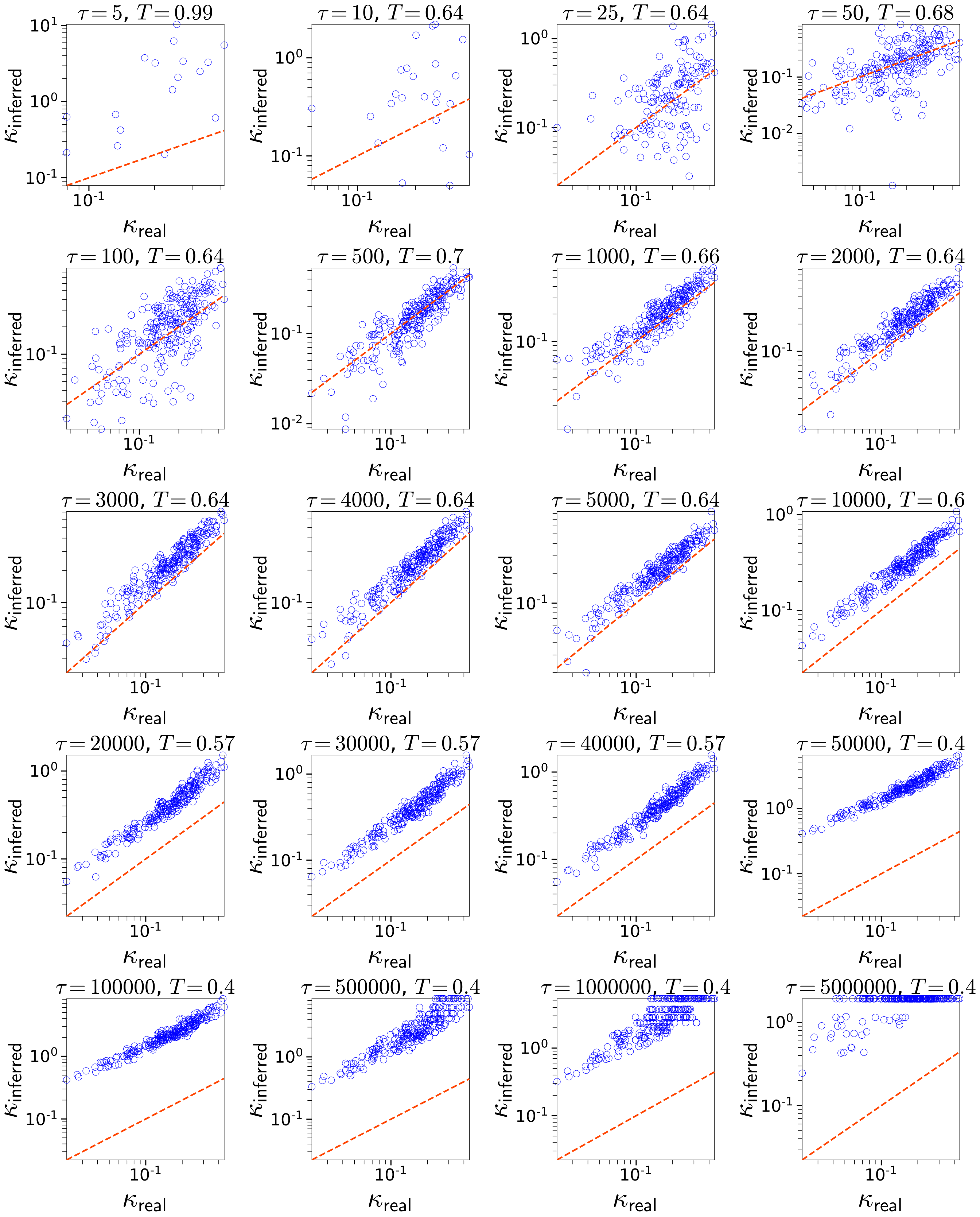

FIG. S19. **Inferred vs. real κ for different aggregation intervals τ.** Same as in Fig. S17 but for the synthetic counterpart of the primary school.

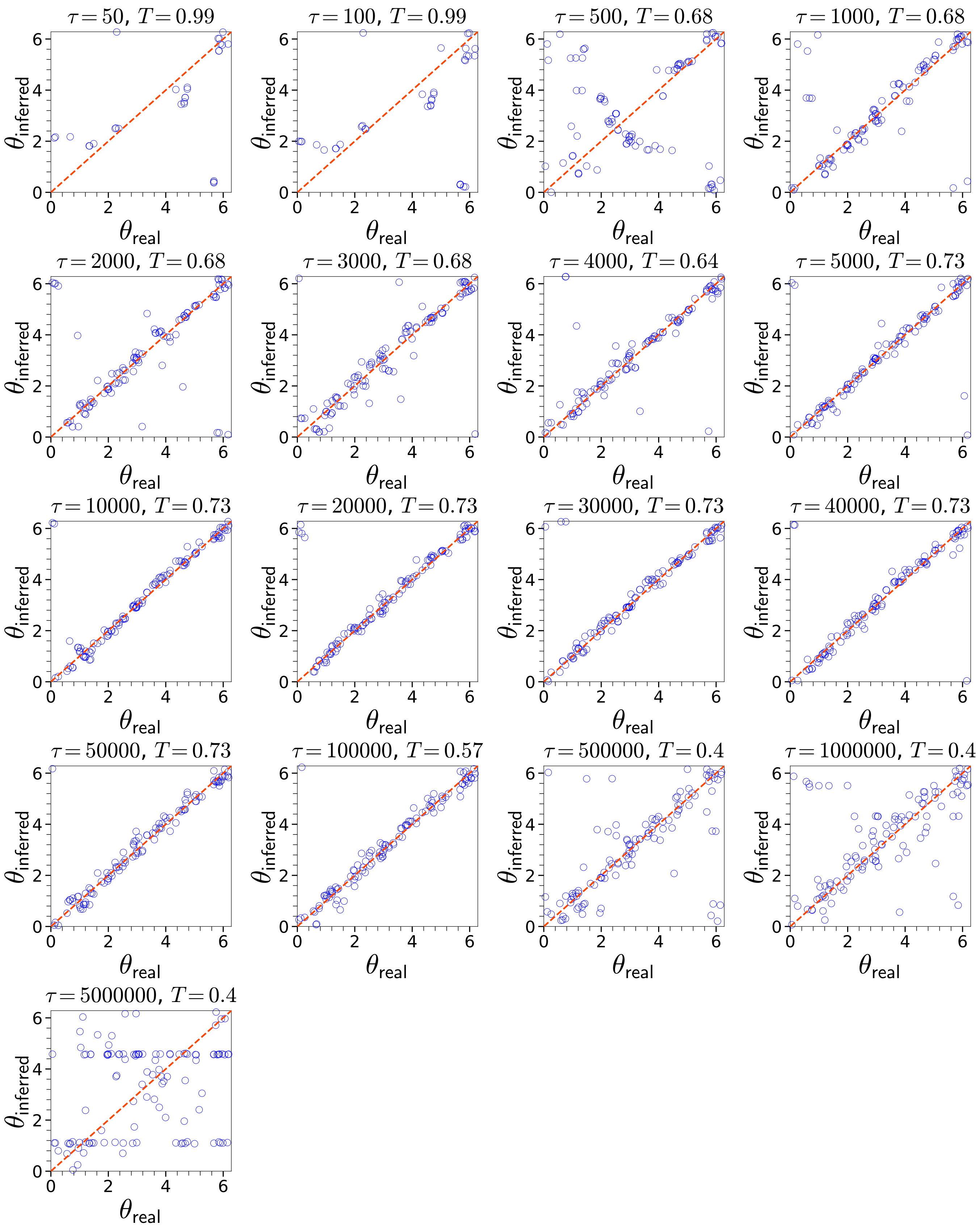

FIG. S20. **Inferred vs. real θ for different aggregation intervals τ.** Same as in Fig. S16 but for the synthetic counterpart of the conference.

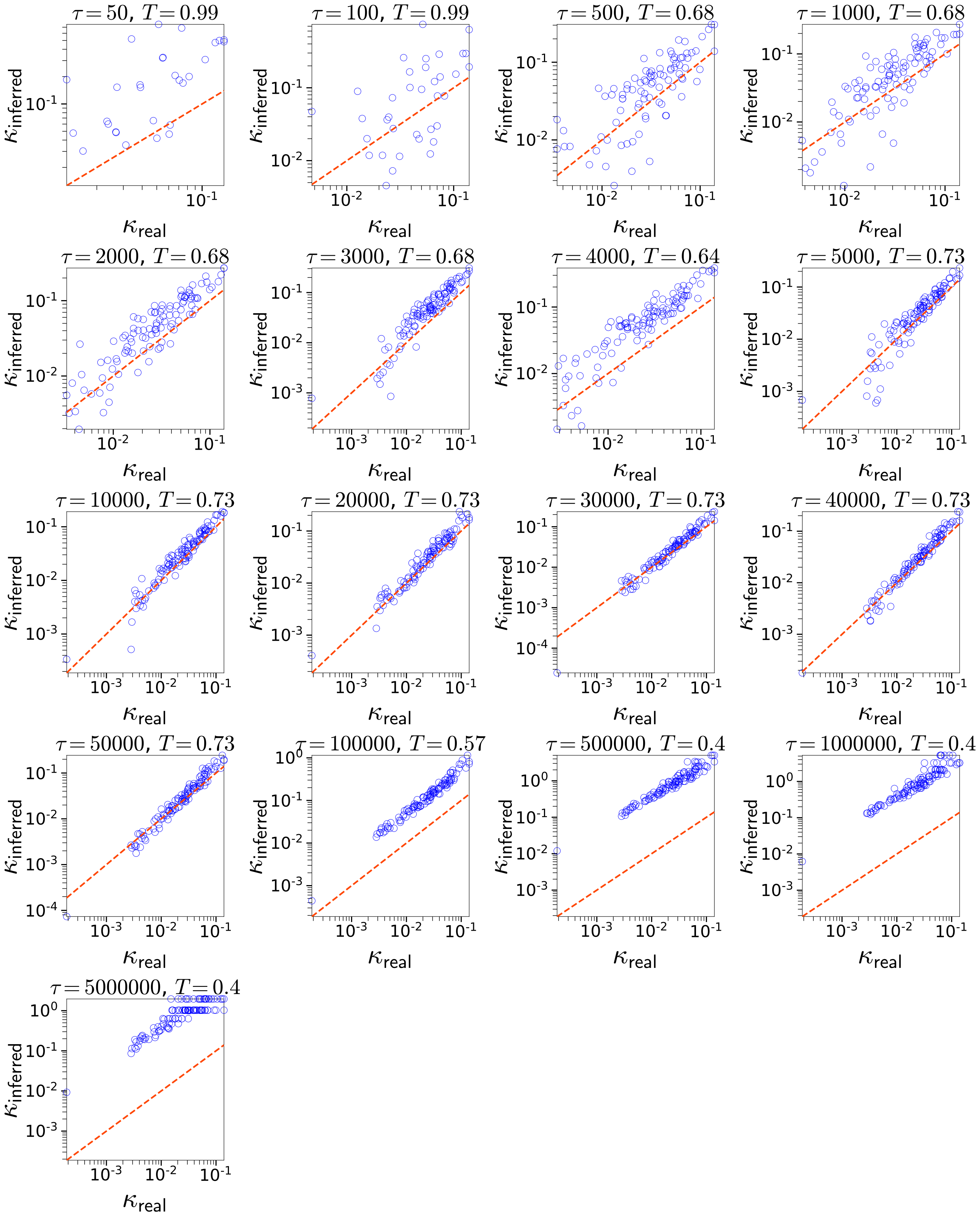

FIG. S21. **Inferred vs. real κ for different aggregation intervals τ.** Same as Fig. S17 but for the synthetic counterpart of the conference.

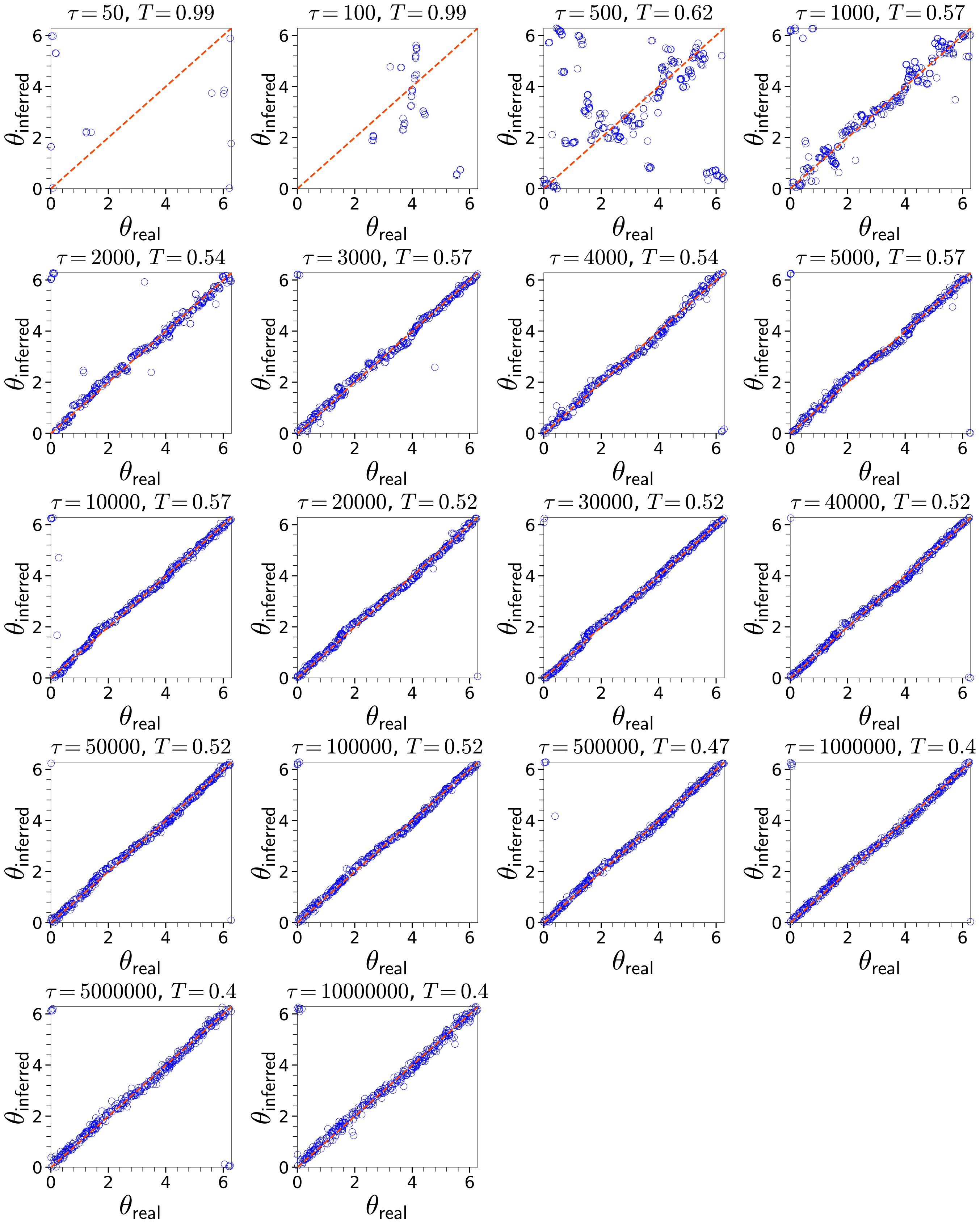

FIG. S22. **Inferred vs. real θ for different aggregation intervals τ.** Same as in Fig. S16 but for the synthetic counterpart of the high school.

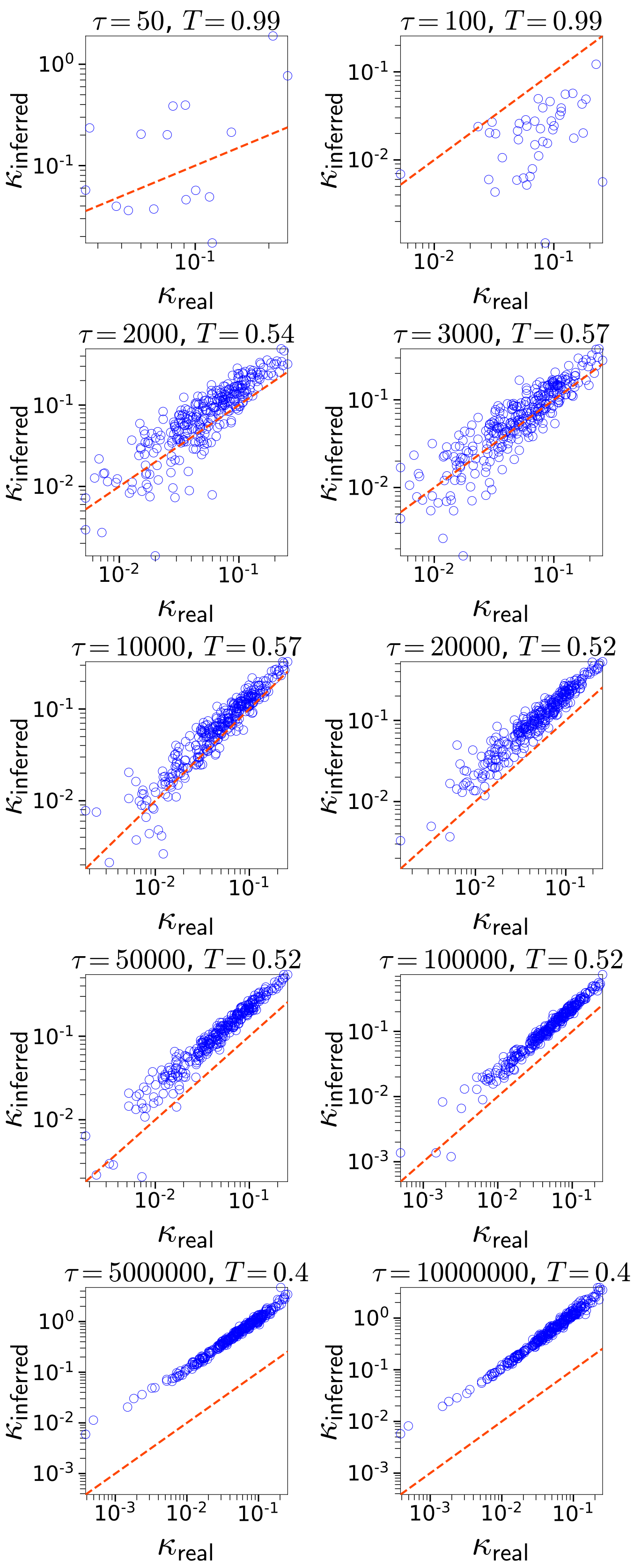

FIG. S23. **Inferred vs. real κ for different aggregation intervals τ.** Same as in Fig. S17 but for the synthetic counterpart of the high school.

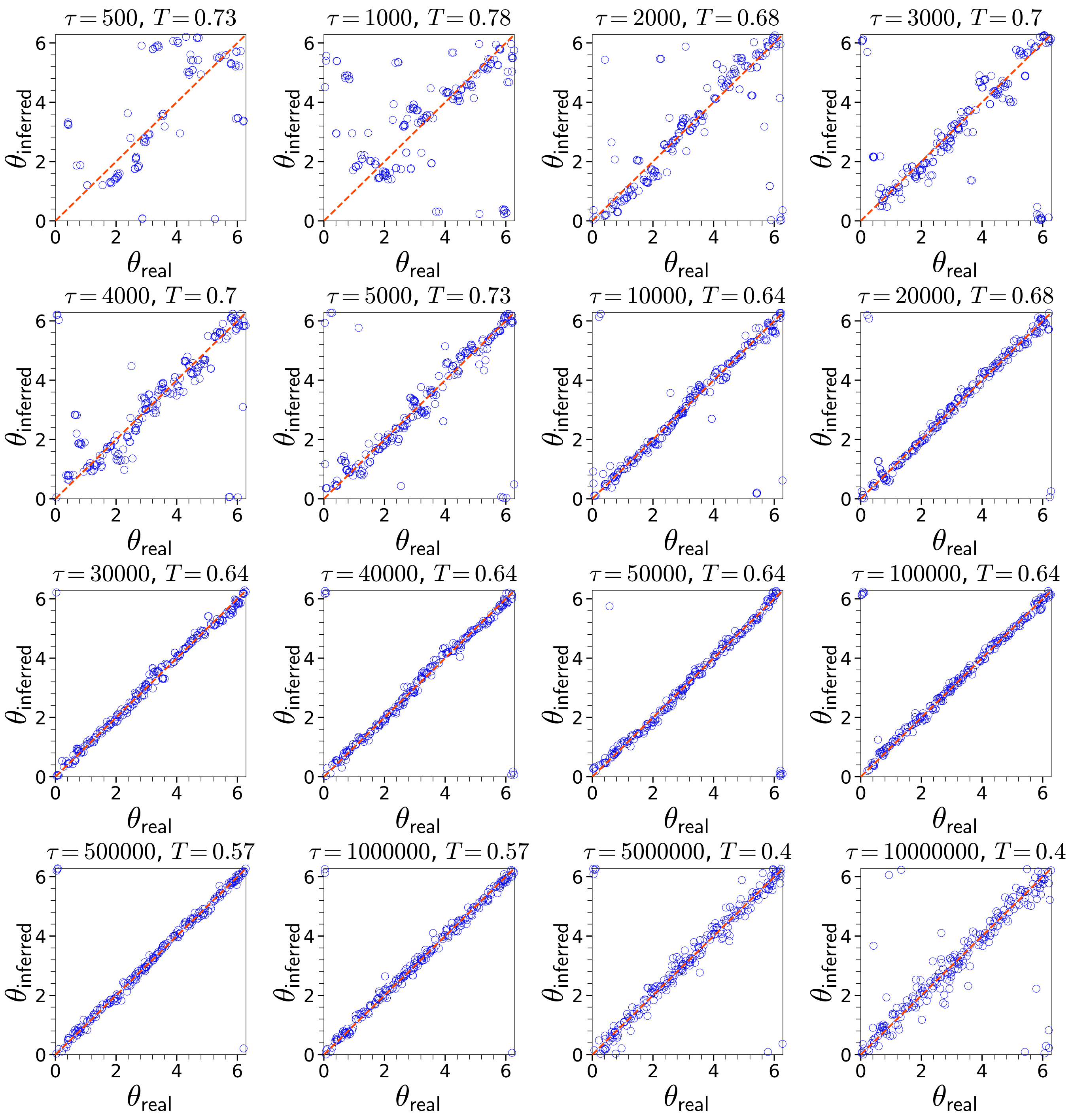

FIG. S24. **Inferred vs. real θ for different aggregation intervals τ.** Same as in Fig. S16 but for the synthetic counterpart of the office building.

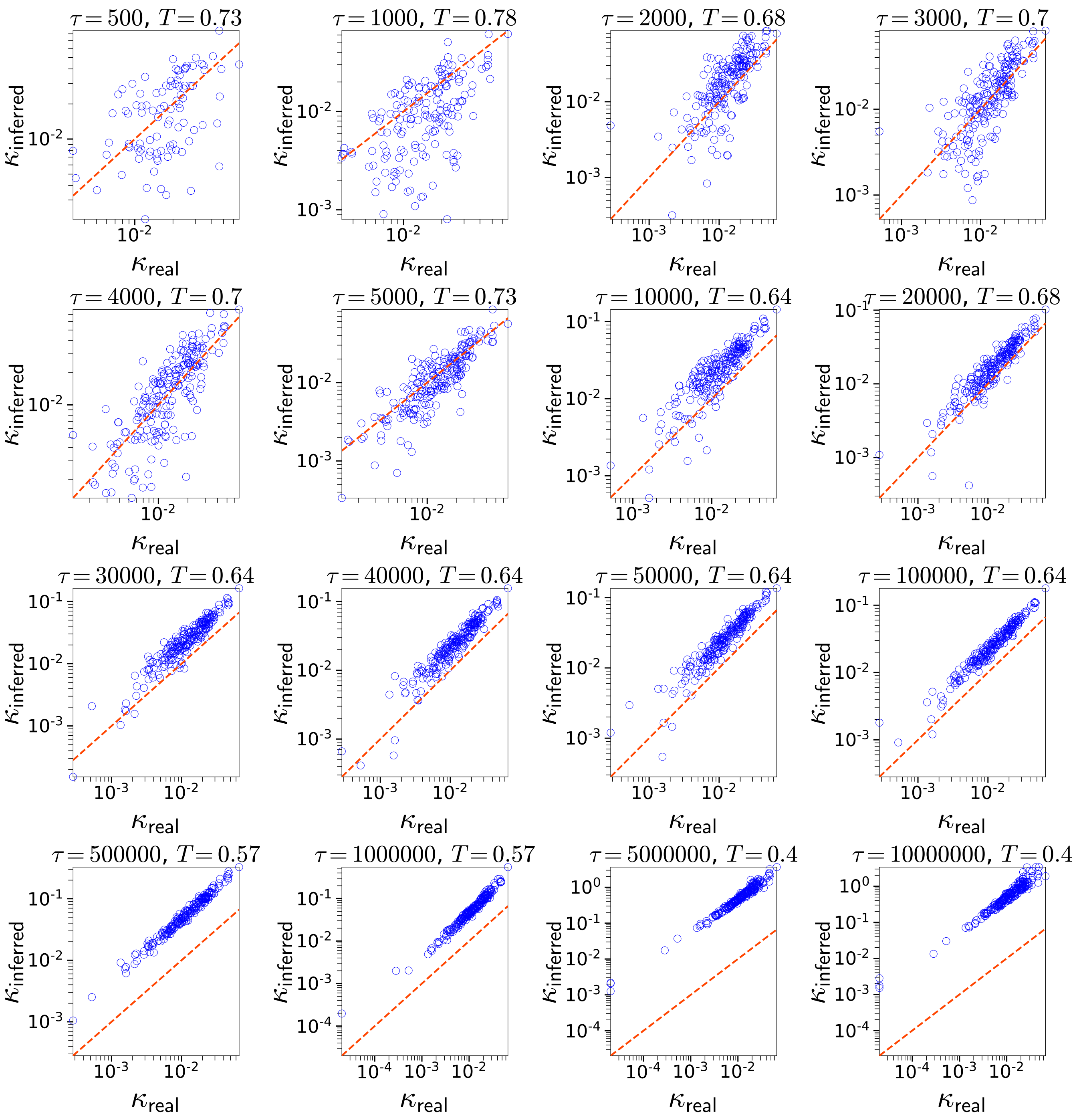

FIG. S25. **Inferred vs. real κ for different aggregation intervals τ.** Same as in Fig. S17 but for the synthetic counterpart of the office building.

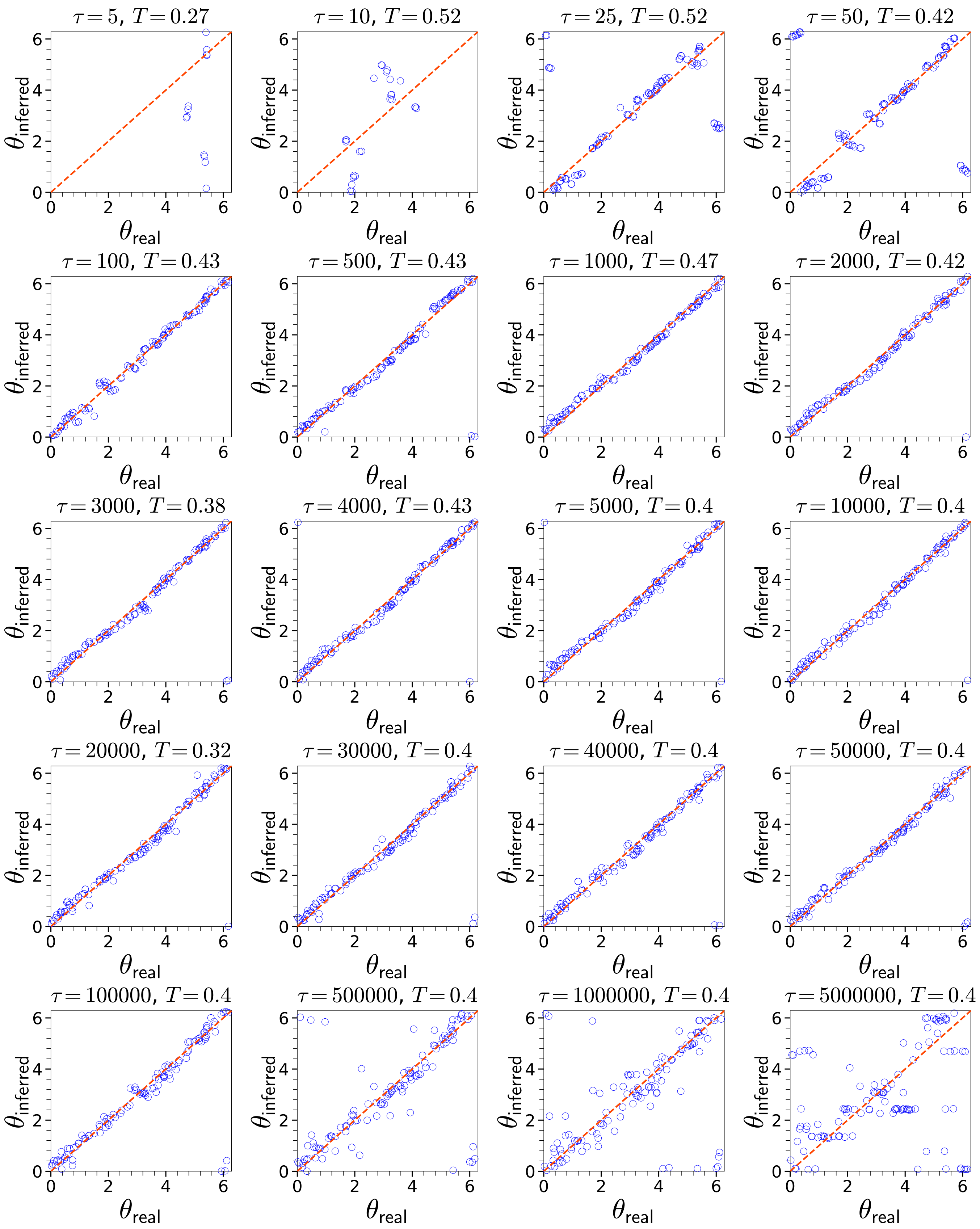

FIG. S26. **Inferred vs. real θ for different aggregation intervals τ.** Same as in Fig. S16 but for the synthetic counterpart of the Friends & Family.

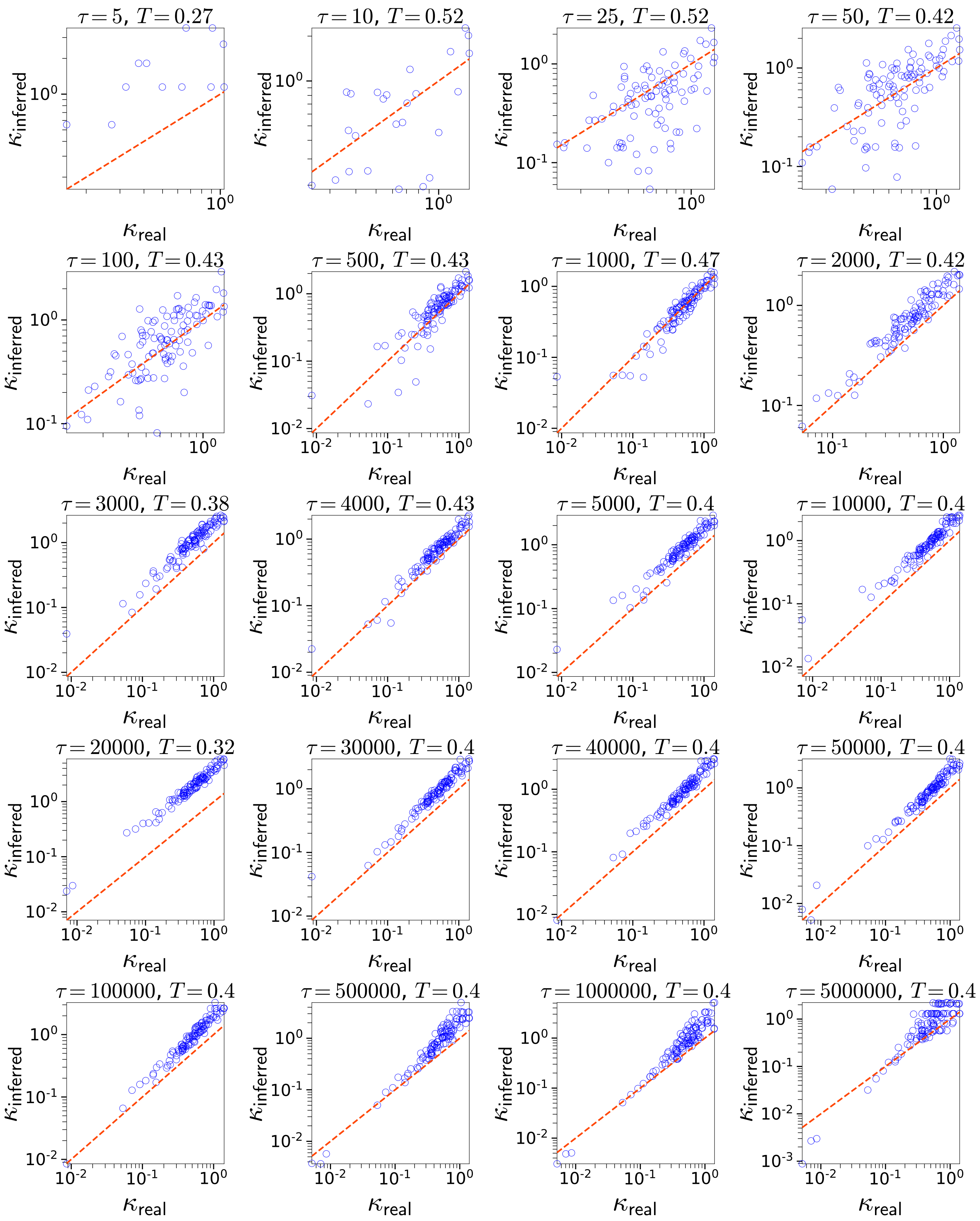

FIG. S27. **Inferred vs. real κ for different aggregation intervals τ.** Same as in Fig. S17 but for the synthetic counterpart of the Friends & Family.
